# Studies on Cement Pastes Exposed to Water and Solutions of Biological Waste

**DOI:** 10.3390/ma15051931

**Published:** 2022-03-04

**Authors:** Agnieszka Sujak, Michał Pyzalski, Karol Durczak, Tomasz Brylewski, Paweł Murzyn, Krzysztof Pilarski

**Affiliations:** 1 Department of Biosystems Engineering, Faculty of Environmental and Mechanical Engineering, Poznań University of Life Sciences, Wojska Polskiego 50 Street, 60-627 Poznań, Poland; agnieszka.sujak@up.poznan.pl (A.S.); karol.durczak@up.poznan.pl (K.D.); krzysztof.pilarski@up.poznan.pl (K.P.); 2 Faculty of Materials Science and Ceramics, AGH University of Science and Technology, A. Mickiewicza 30 Street, 30-059 Kraków, Poland; brylew@agh.edu.pl (T.B.); murzyn@agh.edu.pl (P.M.)

**Keywords:** ordinary Portland cement, biocorrosion, thaumasite, ettringite

## Abstract

The paper presents studies on the early stages of biological corrosion of ordinary Portland cements (OPC) subjected to the reactive media from the agricultural industry. For ten months, cement pastes of CEM I type with various chemical compositions were exposed to pig slurry, and water was used as a reference. The phase composition and structure of hydrating cement pastes were characterized by X-ray diffraction (XRD), thermal analysis (DTA/TG/DTG/EGA), and infrared spectroscopy (FT-IR). The mechanical strength of the cement pastes was examined. A 10 to 16% decrease in the mechanical strength of the samples subjected to pig slurry was observed. The results indicated the presence of thaumasite (C_3_S·CO_2_·SO_3_·15H_2_O) as a biological corrosion product, likely formed by the reaction of cement components with living matter resulting from the presence of bacteria in pig slurry. Apart from thaumasite, portlandite (Ca(OH)_2_)—the product of hydration—as well as ettringite (C_3_A·3CaSO_4_·32H_2_O) were also observed. The study showed the increase in the calcium carbonate (CaCO_3_) phase. The occurrence of unreacted phases of cement clinker, i.e., dicalcium silicate (C_2_S) and tricalcium aluminate (C_3_A), in the samples was confirmed. The presence of thaumasite phase and the exposure condition-dependent disappearance of CSH phase (calcium silicate hydrate), resulting from the hydration of the cements, were demonstrated.

## 1. Introduction

Finding alternative energy sources poses a serious challenge to the scientists dedicated to environmental protection and sustainable development policies [1]. The agricultural industry has now become an excellent source of reagents for the production of biological gases generated from wastes [2].

Waste materials including animal products such as slurry, silage leachate, beet pulp, or manure frequently pose a threat to structural concrete, when in direct contact.

Most reactors used for the production of biological gases using waste from the agricultural industry are made of non-biodegradable plastics.

From the scientific and application point of view, concrete biogas reactors in which energy gases are obtained should be built from cements with increased surface resistance to biological corrosion. This means that extremely durable concretes are required. Ultra-light concretes are yet another alternative for the storage of biological waste. Thanks to its specific microstructure, the migration of aggressive ions that have a destructive effect on the durability of the hardened monolith can be eliminated [3].

At the design stage of concretes resistant to biological corrosion, it should be taken into account that biological gases are obtained through the utilization of organic waste in aerobic (composting) and anaerobic conditions, as well as through methane fermentation processes [4]. The most common biocorrosion of concrete is observed during the storage of maize silage and in the processes of anaerobic waste disposal when leachate is formed during composting. Variable environmental conditions in the contact zone of the deposited material and its reaction with the concrete surface are responsible for biocorrosion. In biological corrosion reactions, microorganisms are a main active factor contributing to the corrosion processes of hardened concrete in various ways [5]. A significant increase in damage to concrete elements (cracks, efflorescence, etc.) can be expected. The main factors influencing a broadly understood biological (chemical) corrosion of concrete are aggressive ions participating in fermentation processes such as SO_4_^2−^. Sulphate ions are formed in the processes of anaerobic decomposition of organic matter from agri-food processing. Their high concentration leads to a deterioration of the structural and mechanical properties of concrete. The main products of sulphate corrosion in hardened concrete are the formed secondary ettringite or brucite responsible for surface cracking, which creates suitable conditions for the penetration of other aggressive ions, for example Cl^−^ [6]. Chloride ions usually exist in the products obtained from agricultural silage. Chloride aggression is based on the reaction of chloride ions with calcium hydroxide, which, in turn, causes a decrease in the pH of the cement paste, the binder in the concrete matrix [7,8]. The next step is the slow carbonation of the cement matrix and consequently, of the concrete [9]. The decrease in pH in concrete will also affect the corrosion of reinforcing steel, which is the basis for the structural properties of hardened reinforced concrete [10]. Thaumasite corrosion is a special case of biological corrosion in hardened concrete mixtures. Carbonation of the cement matrix as a result of the anaerobic respiration of bacteria leads to a decrease in the pH of the concrete matrix and a reaction with sulphate ions under reduced temperature conditions, leading to the disintegration of the CSH phases responsible for the mechanical properties of concrete. In addition, lowering the pH leads to the destruction of the passivation layer of the reinforcing steel and, in turn, to its oxidation and destruction [11]. The occurrence of thaumasite corrosion in the diffraction spectra in samples subjected to biological corrosion was demonstrated in cement pastes kept in pig slurry for a period of 3 months [11]. Research on the development of intelligent additives for Portland cement has shown that dispersed gamma C_2_S can be an interesting additive to Portland cement and can effectively improve the resistance to conditions of exposure to building materials that cause biological corrosion [12]. The most important difference between the gamma and beta C_2_S polymorphs is that the former has no hydraulic properties, while the latter does. The current research and technological works on the gamma C_2_S variety indicate that this phase, when properly activated, has interesting anti-corrosion properties in concrete mixtures [12].

In the presented study, spectroscopic, thermal, and mechanical tests on selected cement pastes made of pure CEM I Portland cements (OPC—ordinary Portland cement), produced in Poland, were carried out. The aim was to determine the possible extent of the early stages of biological corrosion. The samples of cement pastes were exposed to aqueous solutions of pig slurry for a period of 10 months. Samples stored in water were used as a reference.

## 2. Materials

Samples of seven Portland cements of the CEM I type (OPC—Ordinary Portland Cement) were selected for the experiment. The chemical analysis of the Portland cements used for studies is presented in Table 1.

Water (pH = 7.3 ± 0.1) of known chemical composition (Table 2) was used as a reference (control). Pig slurry (Tuszyn, Poland) (pH = 7.4 ± 1.5) obtained from the industrial fattening of pigs was used as an active medium for biocorrosion. The chemical compositions of water and the aqueous solution of biological waste are presented in Table 2 and Table 3, respectively.

The Portland cements used in the experiment contained ≥95% of cement clinker and ≤5% calcium sulphate dihydrate as a setting time regulator. Cements with no additives such as fly ash, blast furnace slag, gaize or ground calcium carbonate, which could significantly affect the corrosion process, were used in the experiments.

The cements used in the experiment were characterized by the same strength class, i.e., 42.5 (this means that after 28 days of setting, the standard mortar containing 25% of OCP reaches the strength of 42.5 Mpa). The examined cements had high initial strength, marked with R (>20 Mpa after 2 days). The exceptions were those from the Chełm and Ożarów cement plants, marked with N indicating that the cement had a normal initial strength (>10 Mpa after 2 days). Detailed information on the properties of the cement pastes used in this experiment is described in the PN-EN-197-1 standard.

XRD, thermal analysis, and FTIR measurement samples were formed into cuboids with dimensions of 40 mm × 40 mm × 160 mm, according to the European standard PN-EN 196-1:2016-07 [13]. The water/cement weight ratio was 0.5. The forms with cement pastes were left for 24 h until the initial strengths were obtained, facilitating the maintenance of the nominal shapes of the beams. After the samples had been removed from the molds, the setting process was continued for another 24 h in a climate chamber providing 100% relative humidity. Then, samples were placed in tightly closed containers filled with the biological aggression medium. Respective samples were kept in water and used as a reference.

Samples were vacuum-dried for 12 h and ground in a vibrating mill (FRITSCH, Germany). This procedure was carried out until the powder grains reaching a granulation size ensuring their screening through a sieve with a mesh size of 63 µm (MULTISERW, Brzeźnica, Poland) were obtained.

Powdered samples for FT-IR measurements were dried under vacuum for 1 h just prior to the experiments.

The mechanical strength tests were carried out on cuboid-shaped micro samples with dimensions of 10 mm × 10 mm × 60 mm after the samples had been seasoned for 10 months in water (reference) or in an aqueous solution of pig slurry.

The samples used as controls were marked from 1_1 to 7_1 (series 1), while samples subjected to pig slurry were labelled from 1_3 to 7_3 (series 3).

## 3. Methods

### 3.1. XRD Measurements

The phase composition of the samples was examined using the X-ray diffraction method (XRD). The apparatus was equipped with a power supply stabilizing the operation of the PW 1140/00/60 X-ray tube and a vertical PW 1050/50 goniometer (Philips Research, Eindhoven, The Netherlands). The device was supplied with a vertical Philips X-ray tube with a Cu anode (Kα = 1.54178 Å); a Ni filter was used for the measurements. The XRD apparatus was equipped with a PW 2216/20 “fine focus” X-ray tube with a power of 1.2 kW (the lamp power used was 1 kW, which corresponded to a lamp voltage of 40 kV and a cathode filament current of 25 mA). The diffractometer settings adjustment system allowed for setting a narrow radiation beam, which improved the accuracy of the measurement data.

### 3.2. Thermal Analysis

The cement pastes were tested using the DTA-TG thermal analysis method, coupled with the EGA gas analysis. Measurements were performed with a STA 449F3 Jupiter (Netzsch, Cracov, Poland) device coupled with a QMS 403 C Aëolos quadrupole mass spectrometer (Netzsch, Cracov, Poland). Measurements were carried out in alumina crucibles (Al_2_O_3_), on samples weighing approx. 75 mg, in a synthetic air atmosphere with a flow rate of 40 mL/min and a heating rate of 15 °C/min. The measurements were obtained within the temperature range between 30 °C and 1000 °C. 

On the basis of the TGA measurements of the mass loss in characteristic temperature ranges, the content of the individual components in the tested samples was estimated.

The content of Ca(OH)_2_ and CaCO_3_ in the initial samples after the calculation of their fraction on the basis of the value of the mass loss on the TG curves was determined from the following relationship:

X = ΔM_TG_ × S (1)

where: X—content of the component in the sample, expressed in % (m/m), ΔM_TG_—the mass loss based on the TG curve in the temperature range characteristic for a given component, expressed in % (m/m), S— stoichiometric coefficient resulting from the chemical composition of a given component.

The content of Ca(OH)_2_ was calculated using the value of S = 4.12 and the content of CaCO_3_ using the value of S = 2.2.

### 3.3. Compressive Strength of Cement Pastes

The changes in the mechanical strength of the cement pastes produced with CEM I after storage in aqueous solutions of biological waste were examined. The tests of compressive strength were performed on a compact-line hydraulic press (Controls S.p.A., Milan, Italy). 

### 3.4. FT-IR Measurements

Fourier transform infrared spectroscopy (FT-IR) was used for determining the transformation of the cement paste structures during the storage in the liquid pig slurry for a period of 10 months.

A Nicolet iS50 FTIR spectrometer (Thermo Scientific, Madison, WI, USA) equipped with a diamond attenuated total reflectance attachment (GladiATR attachment, PIKE Technologies, Madison, WI, USA) was used to collect spectra between 4000 cm^−1^ and 500 cm^−1^, with a nominal resolution of 4 cm^−1^. To obtain an optimal signal-to-noise ratio, 64 scans were collected under the “atmospheric correction” mode. Each spectrum was baseline-corrected using OMNIC software (version 8.2, Thermo Fischer Scientific Inc., Madison, WI, USA). The analysed spectra were averaged over three registered spectra series. Spectral analyses were carried out using GRAMS AI Spectral Notebase (Thermo Fisher Scientific, Madison, WI, USA). Graphs were prepared using Grapher 4 (Golden Software, LLC, Golden, CO, USA).

## 4. Results and Discussion

### 4.1. Phase Composition, Micro-Structure

**Figure 1:**
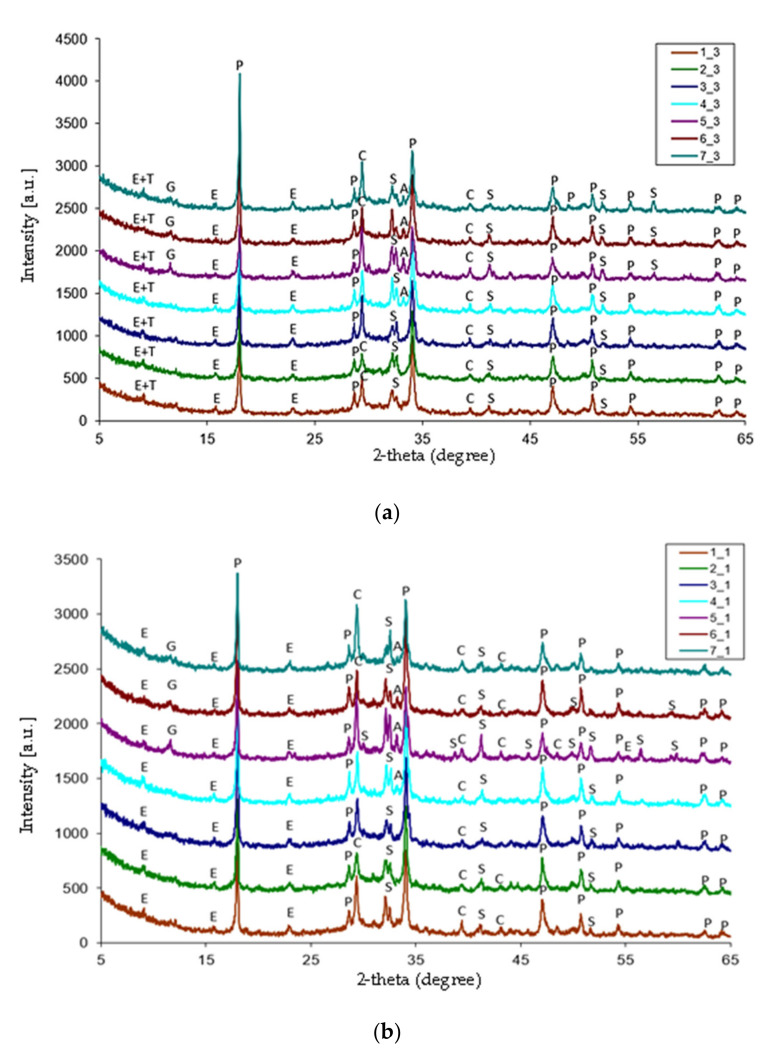
X-ray diffractograms of cement pastes obtained after 10 months of exposure to water (**a**) or to a pig slurry (**b**): T—C_3_S·CO_2_·SO_3_·15H_2_O, E—C_3_A·3CaSO_4_·32H_2_O, P—Ca(OH)_2_, C—CaCO_3_, S—C_2_S, and G—CaSO_4_·2H_2_O.

Figure 1 shows the X-ray diffraction patterns of two sets of cement paste samples exposed to water (the upper panel) and pig slurry (the lower panel), respectively. The conditions of the X-ray measurements for all tested samples were identical. Preliminary evaluation of the X-ray diffraction patterns indicated the presence of a series of reflections of varying intensity, usually attributed to crystalline phases. In addition, a characteristic elevation of the background of the diffraction patterns in the range of the deflection angles 25–40 2-theta (degree) was observed, which may suggest the existence of an amorphous phase in the examined samples.

The portlandite phase, with the composition of Ca(OH)_2_ and calcite (CaCO_3_), was found in both the reference and biological corrosion samples. Depending on the exposure conditions of the tested cement pastes, the diffraction patterns show clear changes in the relative intensity of the reflections coming from these phases, confirming the variable ratio of the mass fraction of portlandite to calcite. In samples immersed in pig slurry, there is a noticeable decrease in the intensity of reflections attributed to the portlandite phase, with a simultaneous increase in the intensity of the peaks characteristic of calcite. The above trend of changes in the intensity of reflections may indicate a reaction between calcium hydroxide and carbon dioxide, which is secreted by bacteria during anaerobic respiration. During this physiological-physicochemical process, a synergy effect takes place, with the participation of dead matter (cement) and living matter (bacteria).

All the diffractograms show a significant increase in the background intensity, presumably due to the presence of CSH phase, which is a product of calcium silicate hydration. Depending on the corrosion conditions, some differences in the intensity of the background are observed. The X-ray diffraction patterns of the surfaces of the samples exposed to animal waste show a slight background flattening in the range of deflection angles 25–40 2-theta (degree). This phenomenon can be associated with the corrosion process during which the decomposition of CSH phase takes place with the formation of thaumasite, under the conditions of an excess of carbonate ions and a reduced reaction temperature. The course of this process was confirmed in all samples subjected to biological corrosion where thaumasite with a small mass fraction can be identified. Moreover, low-intensity reflections appeared on the X-ray diffraction patterns of samples 5_1, 6_1, 7_1, and 4_3, 5_ 3, 6_3, as well as 7_3, indicating the presence of unreacted crystalline phases in these cement pastes in the form of tricalcium aluminate and dihydrate gypsum. In all the tested samples, the fraction of the unreacted clinker phase of the β-C_2_S type, which inherently undergoes slow hydration, was also confirmed. The comparison of the obtained XRD tests with those performed previously [8] showed subtle, but significant differences in the phase composition of the tested cement pastes. Apart from thaumasite, in all samples, the presence of the secondary ettringite phase was demonstrated, which in theory, should not be formed in the hydrating cement pastes after 10 months of the hydration process. Such a phenomenon leads to the conclusion that the formation of subsequent portions of the corrosion product, such as thaumasite, is possible in the later stages of the experiment. The presence of variable amounts of calcium carbonate in slurry-exposed cement pastes is worthy of notice. The comparison of reference samples with samples interacting with living matter, i.e., bacteria, results in an increase in the amount of calcium carbonate phase as a product of Ca(OH)_2_ carbonation during the anaerobic respiration of living matter, i.e., bacteria from animal waste.

### 4.2. Thermal Analysis

**Figure 2:**
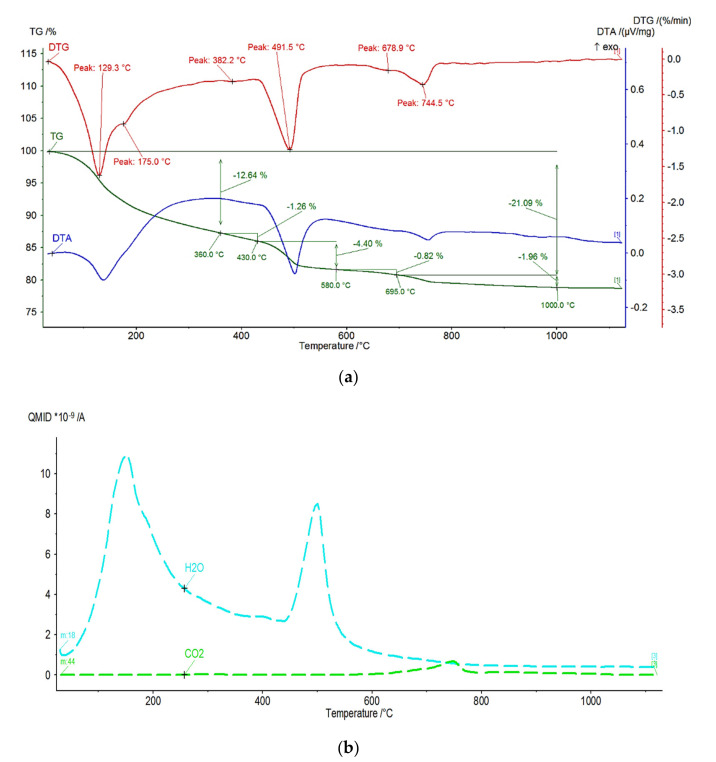
Typical DTA/TG/DTG/EGA thermal curves (**a**) and the respective temperature dependent ion current for H_2_O and CO_2_ release, as indicated (**b**). For the additional data, see Supplementary File.

Figure 2 shows the exemplary DTA/TG/DTG/EGA thermal curves (sample 1_1 selected). All tested samples showed a similar course of thermal curves in terms of quality, and the existing differences concerned the content of individual components. It can be noted that the thermal processes characteristic for this type of material take place in three temperature ranges.

The first temperature range from room temperature (RT) to approx. 300 °C concerns the release of chemically unbound water, the dehydration of gypsum and ettringite, and the dehydration of the CSH phase, which is manifested by a mass loss on the TG curve, accompanied by an endothermic effect on the DTA curve. The second temperature range starts from approx. 450 °C and ends at approx. 550 °C, with the visible mass loss on the TG curve and the endothermic effect on the DTA curve. It includes processes related to the thermal decomposition of portlandite by the dehydroxylation process.

The above thermal changes are related to the release of H_2_O to the atmosphere. The third range characterizes the processes taking place in the temperature range from approx. 650 °C to approx. 1000 °C, visible on the TG curve as a loss of mass, accompanied by the endothermic effect on the DTA curve, related to the thermal decomposition of carbonate resulting in the release of CO_2_. The thermal decomposition of carbonate takes place in the temperature range from approx. 650 °C to approx. 1000 °C. Two stages can be observed, indicating the presence of two forms of carbonate. The first takes place in the temperature range of 580–695 °C, with a lower value of mass loss, and the second in the range of 695–1000 °C.

Table 4 presents the mass loss obtained from the TG curves of the tested samples in the characteristic temperature ranges associated with the thermal processes such as dehydration, dehydroxylation, and decarbonatization.

Table 5 shows the content of Ca(OH)_2_ and CaCO_3_ in the samples calculated on the basis of the mass loss changes from the TG curves.

The analysis showed the decrease in the fraction of portlandite and an increase in the amount of calcite in the samples exposed to pig slurry compared to the samples kept in water. The likely cause of this phenomenon is the secondary carbonation of calcium ions derived from the calcium hydroxide by carbon dioxide released of the bacteria present in pig slurry as a result of their anaerobic respiration. Therefore, thermal DTA/TG/DTG results showing a quantitative change in secondary calcium carbonate, supplement the qualitative XRD tests.

### 4.3. Changes in Compressive Strength of Cement Pastes

**Figure 3:**
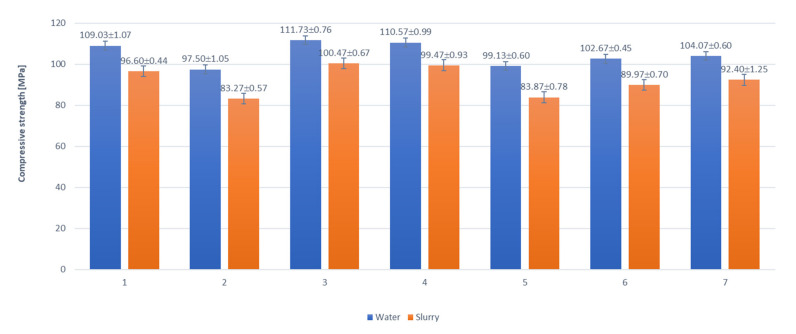
The mean compressive strength (*n* = 6) after 10 months [ Mpa] of cement pastes 1–7 ±S.D. Samples exposed to water (reference)—dark grey; samples exposed to biological corrosion—light grey. X-axis—cement pastes (1–7) subjected to compressive strength tests. Y-axis—values of compressive strength [Mpa].

The results of the experiments on the compressive strength of cement pastes are presented as bar charts in Figure 3. The analysis of the test results shows that all samples of the cement pastes kept in water reached the compressive strength of 100 Mpa or higher. The samples of the cement pastes exposed to pig slurry (under biological corrosion conditions) reached compressive strength values ranging from 80–99 Mpa. All the values obtained were 10% to 16% lower than those of the control samples (stored in water). The decrease in the mechanical strength of the samples subjected to biological waste can be explained by the surface corrosion of the samples resulting from biological aggression, which contributed to the reduction of the surface coherence of the cement paste matrix made up of hydrated calcium silicates (C-S-H).

In the samples stored in the biological medium, both in the phase composition and thermal tests, the increased contents of calcium carbonate were observed. The formation of calcium carbonate, on the one hand, seals the surface of the cement paste samples, but on the other hand, may also reduce their strength parameters. Therefore, it can be concluded that the fraction of open pores on the sample surface, in which a deeper penetration of the corrosive medium takes place, may result in the formation of hydration products that reduce the mechanical parameters of the sample.

### 4.4. FT-IR Analysis

**Figure 4:**
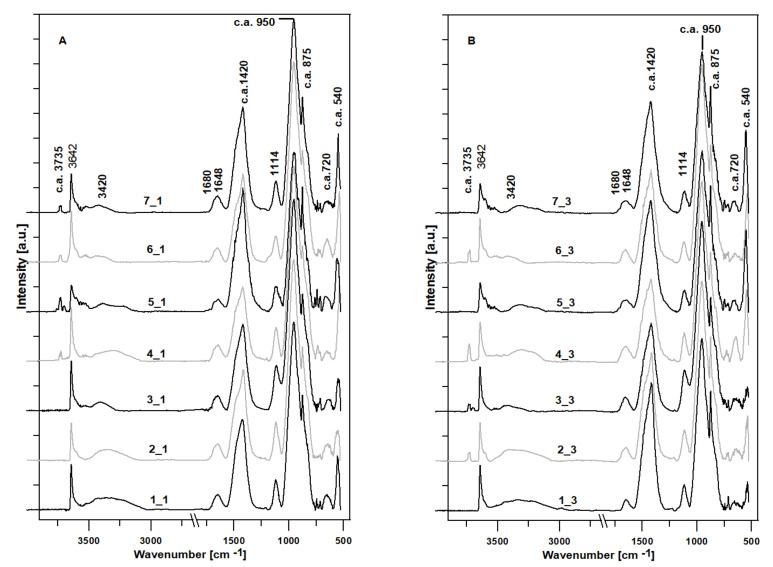
The surface-normalized FTIR spectra of the examined cement pastes in the range of 4000–500 cm^−1^. Cement pastes kept in water (Panel (**A**), series 1), and samples kept in a pig slurry (Panel (**B**), series 3) for a period of 10 months.

Figure 4 shows the FTIR spectra (registered between 4000 cm^−1^ and 500 cm^−1^) of the examined cement pastes. All the samples showed very similar spectroscopic patterns.

The spectral band with the maximum absorption above 3700 cm^−1^ is characteristic of the axial deformation of OH in Si-OH (references 4_1, 5_1, 6_1, and 7_1 and samples 2_3, 3_3, 4_3, and 6_3, subjected to pig slurry). It can be also attributed to the isolated silanol groups (-Si-O^−^…H^+^) [14].

Bands approaching 3650 cm^−1^ represent the OH bending modes, while those closer to 3600 cm^−1^ represent the stretching modes of this group [15]. The whole spectroscopic region between 3600–3000 cm^−1^ is attributed to the hydroxyl ν(O–H) stretching modes. Bands positioned between 3580 cm^−1^ and 3045 cm^−1^ mainly represent the OH stretching in absorbed water. The band with the absorbance maximum at ~3425 cm^−1^ is probably related to the surface OH participation of hydrogen bonds [14] (visible for all the samples). When accompanied by a 1675 cm^−1^ band (a very weak absorption band in samples 4_1 and 5_1, 5_3 and 7_3), it represents the stretching and bending frequencies of water [15,16]. Water is also represented by a shoulders at ~3230 cm^−1^ and at ~1630 cm^−1^ [14]. A very sharp band with a maximum positioned at the wavelengths between 3644 cm^−1^ and 3641 cm^−1^ (in this study, usually at 3642 cm^−1^) most likely represents a –OH stretching vibration in portlandite Ca(OH)_2_—visible in all the examined samples [17,18]. Interestingly, this is more intense and sharper in samples from series 1 as compared to series 3 (except sample 5_3), indicating the decrease in portlandite content as also shown by the TGA analysis. Generally, this represents a weak hydrogen-bonded interlayer of water molecules [19]. A relatively low intensity band with a maximum at c.a. 3620 cm^−1^ overlying the portlandite band represents stretching vibrations of the structural OH groups shared by two octahedral atoms, mainly Al [20]—present only in sample 5_3. Sample 1_3 reveals very small intensity peaks with maximum at 2981 cm^−1^ and 2642 cm^−1^; the first can represent a C-H stretch, possibly in Si-CH_3_ [21], while the second—an -OH stretch in CH_2_OH(CO) [21,22] or a Si-H stretch in the case when the Si atom is bonded both to oxygen and to another Si atoms [23]. Another possibility is that the band at 2981 cm^−1^ represents a -CO vibration of CaCO_3_. As it is accompanied by the sharpening of the peak at 873 cm^−1^, this may indicate the process of sample carbonation [19]. 

In some cases, the next band found between 1700 cm^−1^ and 1550 cm^−1^ consists of two slightly visible modes (as seen for sample 4_1, 5_1, and 5_3) which are the bending modes of water δ-OH, with the maximum positioned at c.a. 1635 cm^−1^ [24,25] superposed with the C-O bending mode, with maximums between 1648 cm^−1^ and 1640 cm^−1^ [26].

An intensive group of peaks located between 1550 cm^−1^ and 1350 cm^−1^ is characteristic of the C-O asymmetric stretching modes, mainly in CaCO_3_ [24]. This spectral region can also contain S-O stretching mode at c.a. 1430 cm^−1^ [27], characteristic of gypsum and ettringite [18,28]. The bands in the range of 1419–1416 cm^−1^, 874 cm^−1^, and 713 cm^−1^ are attributed to the CO_3_^2-^ group. The FTIR spectroscopy highlighted the presence of CaCO_3_ (3 distinct bands at 1414–1418 cm^−1^, 872–876 cm^−1^, and 710–730 cm^−1^). The detection of CaCO_3_ can be assigned to the carbonation process or to the presence of limestone. The presence of CaCO_3_ is mainly a result of the atmospheric CO_2_ absorption during sample hydration [25]. The characteristic vibration modes of the carbonate ion lie approx. at 745–698 cm^−1^ (ν_4_), 910–842 cm^−1^ (ν_2_), 1085–1070 cm^−1^ (ν_1_), and 1590–1425 cm^−1^ (ν_3_). There are also combination bands such as ν_4_ + ν_1_ at 1800 cm^−1^, ν_4_ + ν_3_ at c.a. 2500 cm^−1^, and 2ν_3_ at 2900 cm^−1^ [26].

Silica presents a characteristic region of peaks from 1250 cm^−1^ to 700 cm^−1^ that can provide structural characteristics of the network. Bands between 1250 cm^−1^ and 1000 cm^−1^ correspond to the asymmetric ν(Si-O-H) modes. The Si-O(H) bond stretching appears at ~950 cm^−1^. The symmetric mode of the ν(Si-O-Si) band is found at ~791 cm^−1^, while the Si–O− rocking mode was observed at ~540 cm^−1^ [14]. The presence of the asymmetric stretching of the silicon-oxygen-silicon bond is indicated by a weak band at 1074 cm^−1^ [27].

In most of the samples, the intensity ratio of the main peaks to the maximum at c.a. 950 cm^−1^, representing valence fluctuations of the Si-O bond characteristic, for the C-S-H phase [15], and that at c.a. 1420 cm^−1^, C-O, mainly in CaCO_3_, alters. This ratio decreases in the case of samples 1 (from 2.03 to 1.34), 3 (from 2.38 to 2.15), and 4 (from 2.26 to 2.19), most likely indicating the decrease in C-S-H phase, accompanied by the increase in CaCO_3_. The increase in this ratio is observed for samples 2 (change from 1.94 to 2.07) and 5 (from 1.25 to 1.42), while it is stable for sample 6 (2.2). This may indicate different processes occurring during the storage of samples in the pig slurry, guided by a slightly different phase composition of samples.

The main characteristic peaks of C-S-H phase, which is the primary binding phase in hydrated Portland cement, are located in the range between 1020–970 cm^−1^ [26]. C-S-H peaks at 970 cm^−^^1^ and 870 cm^−^^1^ could be due to the stretching vibration of the Si-O groups. Consequently, the peak at 670 cm^−^^1^ is assigned to the bending vibration of Si-O-Si. Shorter maximum wavenumbers can represent the Al-O-Si stretch at c.a. 700 cm^−1^ and the AlO_6_ stretch and bond at c.a. 600 cm^−1^ [14]. The peak observed around 550 cm^−1^ is characteristic of Fe-O vibrations [29].

**Figure 5:**
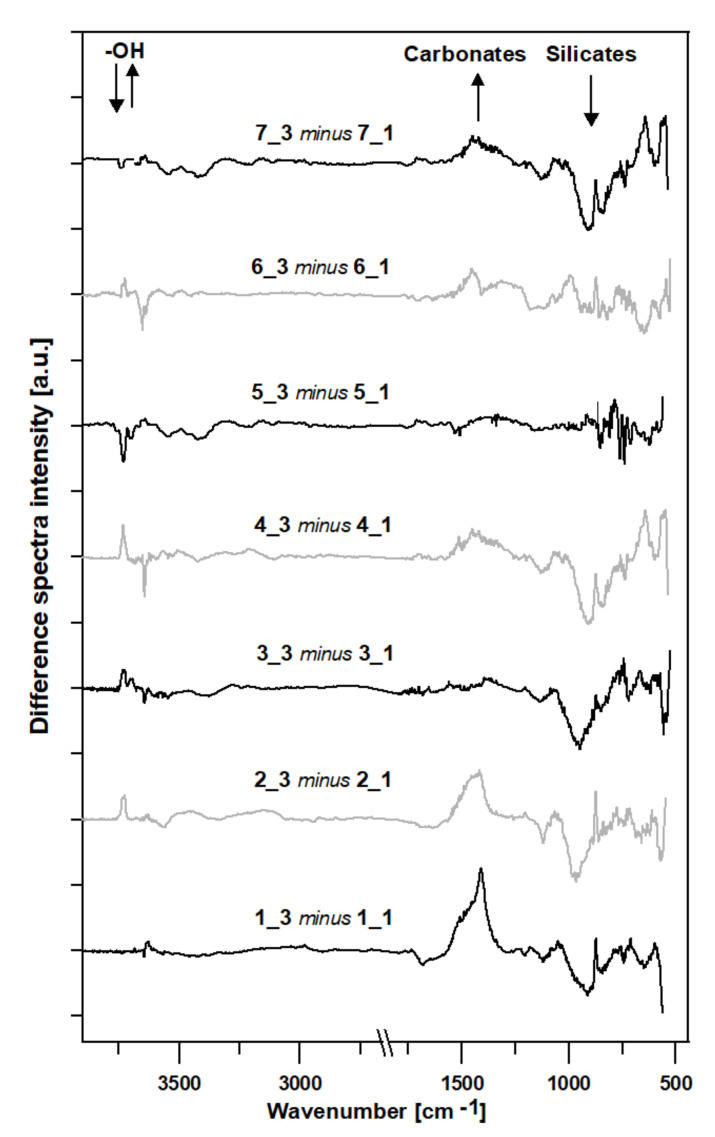
The differences in the spectra in the range of 4000–500 cm^−1^, where the respective absorbance spectra of the cement pastes submerged in water were subtracted from the absorbance spectra of the cement pastes stored in pig slurry for 10 months. The directions of the changes in the spectroscopic regions are indicated by arrows.

The slurry-mediated reorganization of the cement paste structures can be additionally studied by the analysis of the differential spectra (Figure 5), where the absorbance spectra of the cement pastes stored in water were subtracted from the respective spectra of the cement pastes subjected to pig slurry. As seen, the studied samples respond differently to the slurry-mediated corrosion. Despite this, the overall effects can be considered similar.

For most of the samples, the effect of the increase in the 1500–1300 cm^−1^ region, characteristic of a C-O asymmetric stretching mode specific to CaCO_3_, is accompanied by a decrease in the 1100–800 region characteristic for the primary binding phase in hydrated Portland cement—C-S-H.

Generally, peaks in the spectroscopic region of 1500–1400 cm^−1^ represent carbonates, while peaks located between 1200–900 cm^−1^ represent silicates.

The next spectral area indicating structural reorganization is that found at the high wavenumbers. Sharp peaks above 3640 cm^−1^ indicate stretching vibrations of the surface OH groups, as well as weak hydrogen-bonded interlayer water molecules [15]. the exposure of the samples to pig slurry introduced reorganization within the O-H groups, most likely guided by the formation and/or breaking of hydrogen bonds.

## 5. Conclusions

It is very difficult to analyse the changes which occurred in the samples stored in pig slurry due to their complex nature. However, the following conclusions can be drawn from the research carried out in this paper:

1. The use of different exposure conditions for pastes made of Portland cements (OPC) with variable chemical compositions affects the quantitative and qualitative composition of the tested samples.
2. The following crystalline phases were determined in the phase composition of cement pastes kept in water for a period of 10 months: C_3_A·3CaSO_4_·32H_2_O, Ca(OH)_2_, CaCO_3_, C_2_S, C_3_A, and CaSO_4_·2H_2_O.
3. The cement pastes exposed to biological corrosion conditions, apart from the above-mentioned phases, showed the presence of thaumasite, C_3_S·CO_2_·SO_3_·15H_2_O, as the corrosion product.
4. In samples with thaumasite, the disintegration of the CSH phase and an increase in the content of calcium carbonate, CaCO_3_, were observed.
5. Bacteria present in pig slurry, and the metabolic processes resulting from their anaerobic respiration, increase the content of calcium carbonate, CaCO_3_, in the samples.
6. A decrease in mechanical strength of between 10% and 16% was observed in the samples subjected to pig slurry.

## Figures and Tables

**Table 1:** The chemical composition of OCP (Ordinary Portland Cement).

Oxides/ Ions [%]	Chełm CEM I 42.5N	Rudniki CEM I 42.5R	Górażdże CEM I 42.5R	Ożarów CEM I 42.5N	Odra CEM I 42.5R	Warta CEM I 42.5R	Małogoszcz CEM I 42.5R
	1	2	3	4	5	6	7
SiO_2_	21.70	20.81	21.66	21.56	19.36	22.47	24.27
Al_2_O_3_	3.30	4.69	5.14	4.76	5.75	6.05	4.29
Fe_2_O_3_	4.56	3.77	2.77	3.14	2.83	2.72	2.80
CaO	65.52	65.79	65.36	64.94	66.99	63.67	64.01
MgO	1.12	1.33	1.46	1.92	1.68	1.91	1.10
SO_3_	3.14	2.65	2.52	2.63	2.20	2.12	2.30
K_2_O	0.41	0.77	0.86	0.71	1.05	0.80	1.10
Na_2_O	0.20	0.14	0.15	0.30	0.11	0.20	0.10
Cl^−^	0.05	0.05	0.08	0.04	0.03	0.06	0.03
Total	100.0	100.0	100.0	100.0	100.0	100.0	100.0

**Table 2:** The chemical analysis of water used as a reference.

Parameter	Value	Unit	Research Method	Standard
As	<0.001	mg/L	Flame atomic absorption spectrometry (FAAS)	PN-ISO 8288:2002, method A PN-EN 13346:2002 p. 8.3
NO_3_^−^	1.60 ± 0.03	mg/L	Spectrophotometry; concentration of nitrite nitrogen calculated	PN-EN 26777:1999
CN^−^	<0.005	mg/L	Determination of free, bound, and total cyanides by titration and argentometry	PN-82, C04603/02
F^−^	0.41 ± 0.01	mg/L	Spectrophotometry	PB-8 from 18.06.2020 based on HACH 8029
Mg	14.00 ± 0.11	mg/L	Titration method	PN-ISO 6059:1999
Cu	<0.003	mg/L	Flame atomic absorption spectrometry (FAAS)	PN-ISO 8288:2002, method A PN-EN 13346:2002 p. 8.3
Pb	<0.001	mg/L	Flame atomic absorption spectrometry (FAAS)	PN-ISO 8288:2002, method A PN-EN 13346:2002 p. 8.3
Hg	<0.0001	mg/L	Flame atomic absorption spectrometry (FAAS)	PN-ISO 8288:2002, method A PN-EN 13346:2002 p. 8.3
SO_4_^²−^	175.0 ± 12.1	mg/L	Weight method	PN-ISO 9280:2002
Total hardnessCaCO_3_ and MgCO_3_	377.0 ± 18.6	mg/L	The total content of calcium and magnesium (general hardness); titration method	PN-ISO 6059:1999
Ca	87.0 ± 1.9	mg/L	Titration method	PN-ISO 6058:1999
Ife	0.20 ± 0.01	mg/L	Spectrophotometry	PN-ISO 6332:2001
Total trihalomethanes THM	3.0 ± 0.5	µg/L	Spectrophotometry	PN-82/C-04576.08
Cl^−^	0.100 ± 0.006	mg/L	Titration method	PN-ISO 9297:1994

**Table 3:** The chemical analysis of an aqueous solution of a biological medium.

Parameter	Value	Unit	Research Method	Standard
N—Kjeldahl total	1240.0 ± 60.4	mg/L	Titration method	PN-ISO 11261:2002
NH_4_^+^	1050.0 ± 51.1	mg/L	Titration method; concentration of ammonium and ammonia calculated	PN-ISO 5664:2002
N—Total nitrogen	1350.0 ± 63.5	mg/L	Calculated	-
NO_2_^−^	0.032 ± 0.003	mg/L	Spectrophotometry; concentration of nitrite nitrogen calculated	PN-EN 26777:1999
NO_3_^−^	0.31 ± 0.05	mg/L	Spectrophotometry; nitrate nitrogen concentration calculated	PN-82/C-04576.08
Cr	0.40 (-) *	mg/L	Spectrophotometry	PN-77/C-04604-08
Cd	0.05 ± 0.01	mg/L	Flame atomic absorption spectrometry (FAAS)	PN-ISO 8288:2002 method A PN-EN 13346:2002 p. 8.3
Ni	0.09 ± 0.01	mg/L	Flame atomic absorption spectrometry (FAAS)	PN-ISO 8288:2002 method A PN-EN 13346:2002 p. 8.3
Pb	<0.5	mg/L	Flame atomic absorption spectrometry (FAAS)	PN-ISO 8288:2002 method A PN-EN 13346:2002 p. 8.3
Hg	<0.003	mg/L	Flame atomic absorption spectrometry (FAAS)	PN-ISO 8288:2002 method A PN-EN 13346:2002 p. 8.3
Ca	68 (-) *	mg/L	Titration method	PN-ISO 6058:1999
Mg	4.37 ± 0.87	mg/L	Calculated	PN-C-04554-4:1999 app. A
P—Total phosphorus	352 (-)	mg/L	Spectrophotometry	PN-EN ISO 6878:2006 pkt.8 +Ap1 2010 +Ap2 2010
K	684.0 ± 15.4	mg/L	Flame atomic absorption spectrometry (FAAS)	PN-ISO 9964-2:1994; PN-EN 13346:2002 p. 8.3
Dry mass	1.31 ± 0.06	mg/L	Weight method	PN-EN 12880:2004

(-)* single measurement.

**Table 4:** The mass loss of the tested samples and characteristic temperature ranges during heating on the basis of the TG curve.

Sample	Temperature Range [°C]			
	Dehydration	Dehydroxylation	Decarbonatization	
	30–360	430–580	580–695	695–1000
1_1	12.64	4.40	0.82	1.96
2_1	13.41	4.21	0.76	0.94
3_1	12.64	4.24	0.83	2.23
4_1	12.90	4.61	0.71	1.76
5_1	11.51	3.36	0.75	2.30
6_1	13.27	4.59	0.75	1.83
7_1	12.44	3.56	0.93	2.50
1_3	13.20	4.41	0.88	2.30
2_3	13.96	4.18	0.83	1.46
3_3	14.09	4.06	0.94	2.75
4_3	12.60	4.49	0.93	2.49
5_3	11.63	3.07	0.99	3.51
6_3	13.71	4.14	1.04	2.94
7_3	13.47	3.42	1.38	2.80

**Table 5:** The quantitative content of portlandite, Ca(OH)_2_, and calcium carbonate, CaCO_3_ [% (m/m)], in the examined samples determined on the basis of the mass loss in the respective temperature ranges.

Sample	Xca(OH)_2_	XcaCO_3_
	% (m/m)	% (m/m)
1_1	18.13	6.31
2_1	17.35	3.86
3_1	17.47	6.95
4_1	18.99	5.61
5_1	13.84	6.92
6_1	18.91	5.86
7_1	14.67	7.79
1_3	18.17	7.22
2_3	17.22	5.20
3_3	16.73	8.38
4_3	18.50	7.76
5_3	12.65	10.22
6_3	17.06	9.03
7_3	14.09	9.49

## Data Availability

Data available on request.

## Supplementary Materials

The following supporting information can be downloaded at: https://www.mdpi.com/article/10.3390/ma15051931/s1, Figure S1: Sample 1-1. DTA/TG/DTG/EGA thermal curve (the upper panel) and the respective temperature dependent ion current for H_2_O and CO_2_ release, as indicated (the lower panel). Figure S2. Sample 2-1. DTA/TG/DTG/EGA thermal curve (the upper panel) and the respective temperature dependent ion current for H_2_O and CO_2_ release, as indicated (the lower panel). Figure S3. Sample 3-1. DTA/TG/DTG/EGA thermal curve (the upper panel) and the respective temperature dependent ion current for H_2_O and CO_2_ release, as indicated (the lower panel). Figure S4. Sample 4-1. DTA/TG/DTG/EGA thermal curve (the upper panel) and the respective temperature dependent ion current for H_2_O and CO_2_ release, as indicated (the lower panel). Figure S5. Sample 5-1. DTA/TG/DTG/EGA thermal curve (the upper panel) and the respective temperature dependent ion current for H_2_O and CO_2_ release, as indicated (the lower panel). Figure S6. Sample 6-1. DTA/TG/DTG/EGA thermal curve (the upper panel) and the respective temperature dependent ion current for H_2_O and CO_2_ release, as indicated (the lower panel). Figure S7. Sample 7-1. DTA/TG/DTG/EGA thermal curve (the upper panel) and the respective temperature dependent ion current for H_2_O and CO_2_ release, as indicated (the lower panel). Figure S8. Sample 1-3. DTA/TG/DTG/EGA thermal curve (the upper panel) and the respective temperature dependent ion current for H_2_O and CO_2_ release, as indicated (the lower panel). Figure S9. Sample 2-3. DTA/TG/DTG/EGA thermal curve (the upper panel) and the respective temperature dependent ion current for H_2_O and CO_2_ release, as indicated (the lower panel). Figure S10. Sample 3-3. DTA/TG/DTG/EGA thermal curve (the upper panel) and the respective temperature dependent ion current for H_2_O and CO_2_ release, as indicated (the lower panel). Figure S11. Sample 4-3. DTA/TG/DTG/EGA thermal curve (the upper panel) and the respective temperature dependent ion current for H_2_O and CO_2_ release, as indicated (the lower panel). Figure S12. Sample 5-3. DTA/TG/DTG/EGA thermal curve (the upper panel) and the respective temperature dependent ion current for H_2_O and CO_2_ release, as indicated (the lower panel). Figure S13. Sample 6-3. DTA/TG/DTG/EGA thermal curve (the upper panel) and the respective temperature dependent ion current for H_2_O and CO_2_ release, as indicated (the lower panel). Figure S14. Sample 7-3. DTA/TG/DTG/EGA thermal curve (the upper panel) and the respective temperature dependent ion current for H_2_O and CO_2_ release, as indicated (the lower panel).
