# Supplementary material for: Studies on Cement Pastes Exposed to Water and Solutions of Biological Waste
Source: Materials (Basel). 2022 Mar 4;15(5):1931. doi: 10.3390/ma15051931 (PMC8911754; doi:10.3390/ma15051931)
Supplement: Supplementary file 1 [file materials-15-01931-s001.zip › materials-1600134-supplementary.pdf]

materials

Supplementary Materials

Studies on Cement Pastes Exposed to Water and Solutions of Biological Waste

Agnieszka Sujak [1], Michał Pyzalski [2,*], Karol Durczak [1], Tomasz Brylewski [2], Paweł Murzyn [2] and Krzysztof Pilarski [1]

Citation: Sujak, A.; Pyzalski, M.; Durczak, K.; Brylewski, T.; Murzyn, P. Studies on Cement Pastes Exposed to Water and Solutions of Biological Waste. *Materials* **2022**, *15*, 1931. https://doi.org/10.3390/ma15051931

Academic Editor: Sara Cattaneo

Received: 31 January 2022
Accepted: 3 March 2022
Published: 4 March 2022

Publisher's Note: MDPI stays neutral with regard to jurisdictional claims in published maps and institutional affiliations.

Copyright: © 2022 by the authors. Licensee MDPI, Basel, Switzerland. This article is an open access article distributed under the terms and conditions of the Creative Commons Attribution (CC BY) license (https://creativecommons.org/licenses/by/4.0/).

DTA/TG/DTG and EGA curves of tested samples:

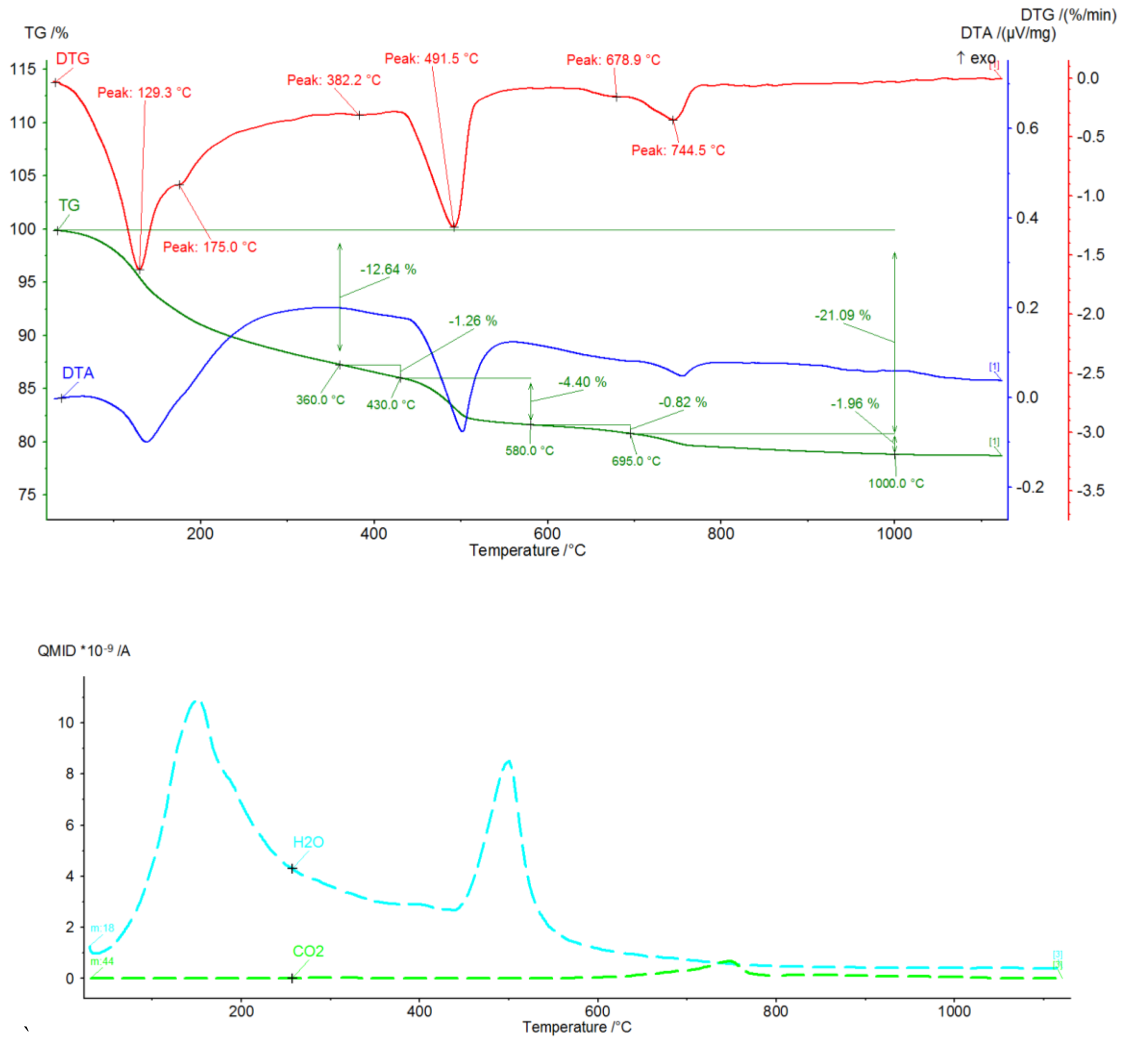

Figure S1. - Sample 1-1. DTA/TG/DTG/EGA thermal curve (the upper panel) and the respective temperature dependent ion current for H_2O and CO_2 release, as indicated (the lower panel).

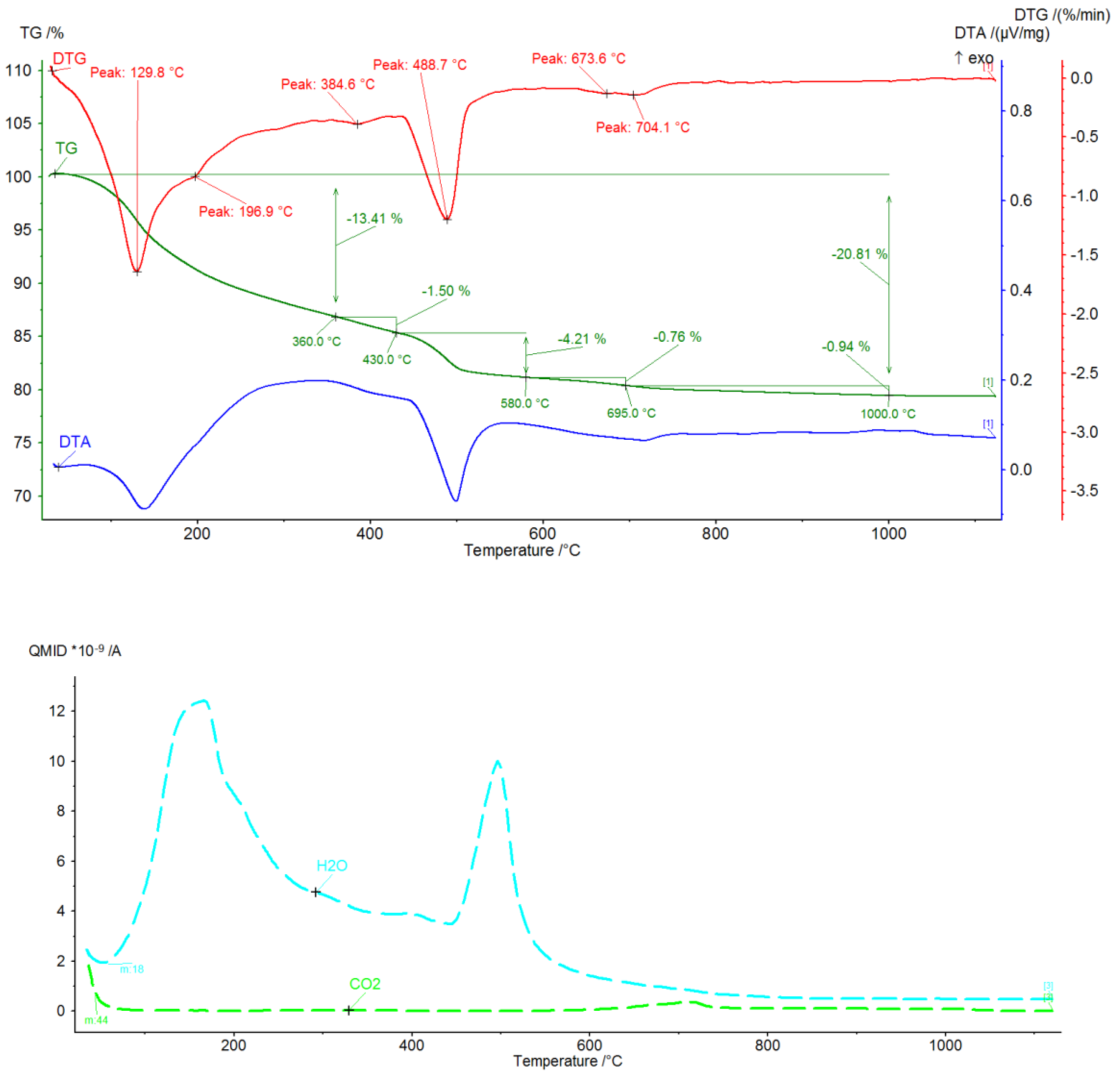

Figure S2. - Sample 2-1. DTA/TG/DTG/EGA thermal curve (the upper panel) and the respective temperature dependent ion current for H_2O and CO_2 release, as indicated (the lower panel).

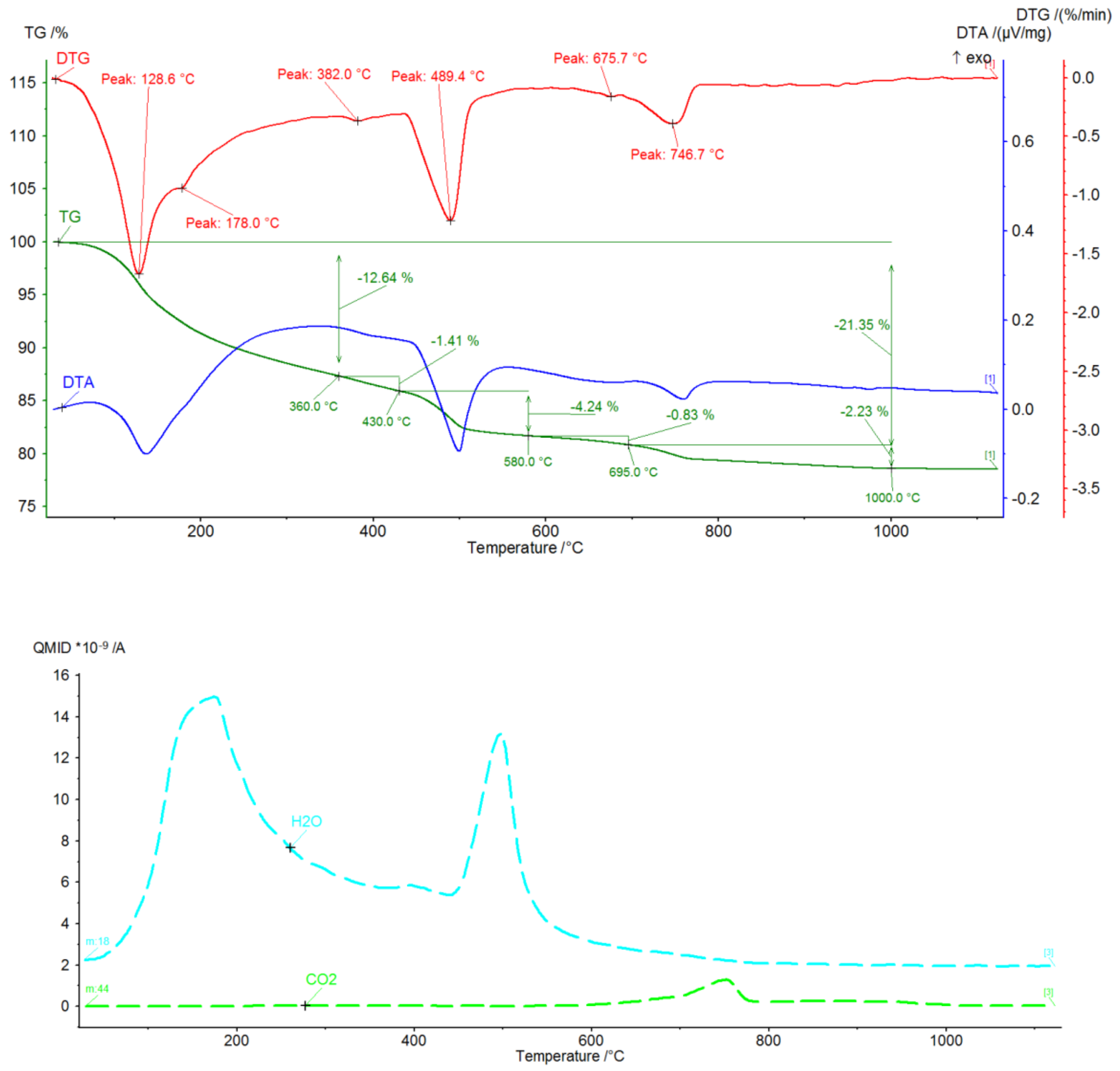

Figure S3. -Sample 3-1. DTA/TG/DTG/EGA thermal curve (the upper panel) and the respective temperature dependent ion current for H_2O and CO_2 release, as indicated (the lower panel).

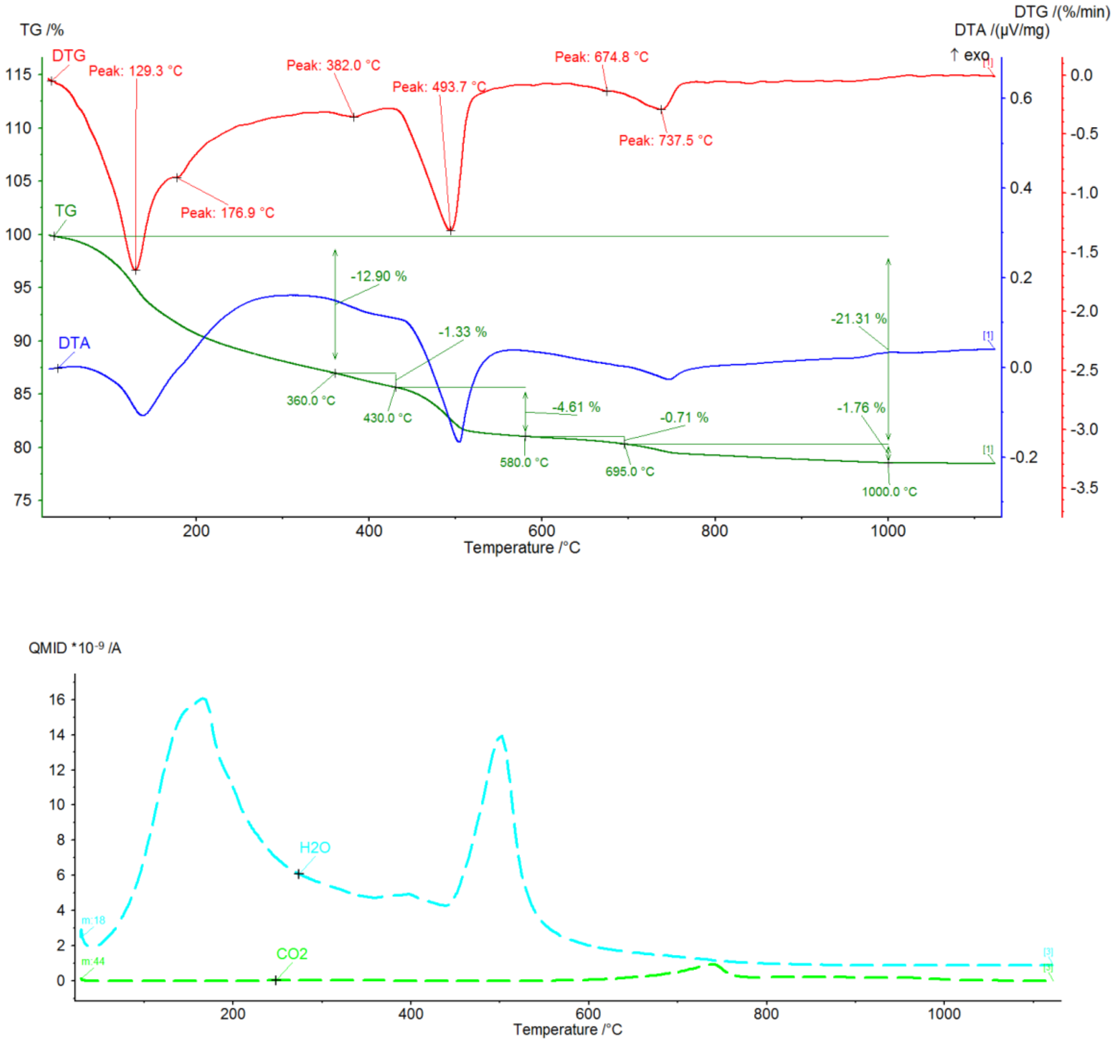

Figure S4. - Sample 4-1. DTA/TG/DTG/EGA thermal curve (the upper panel) and the respective temperature dependent ion current for H_2O and CO_2 release, as indicated (the lower panel).

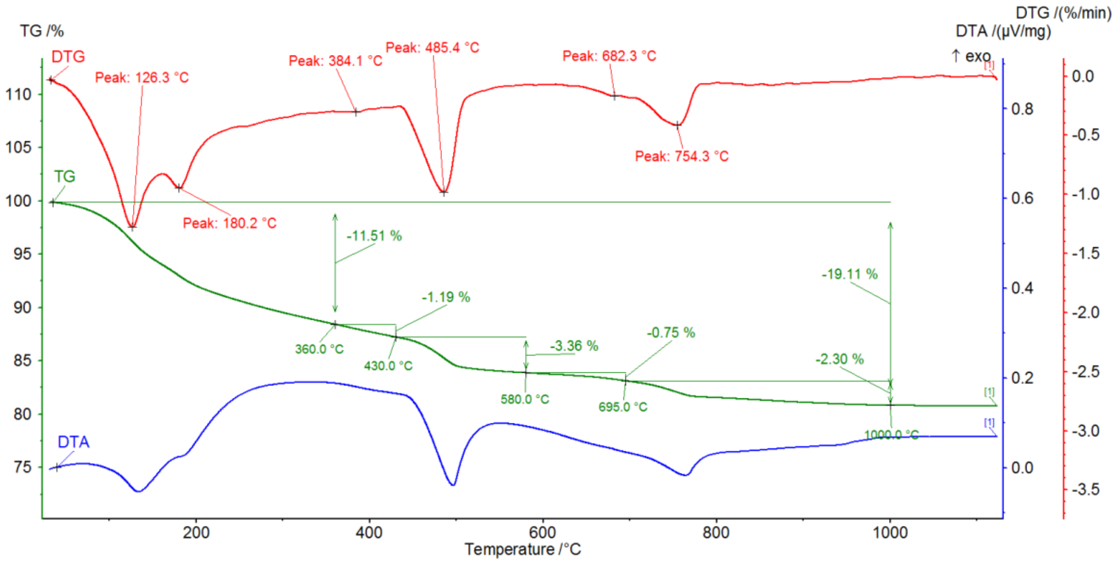

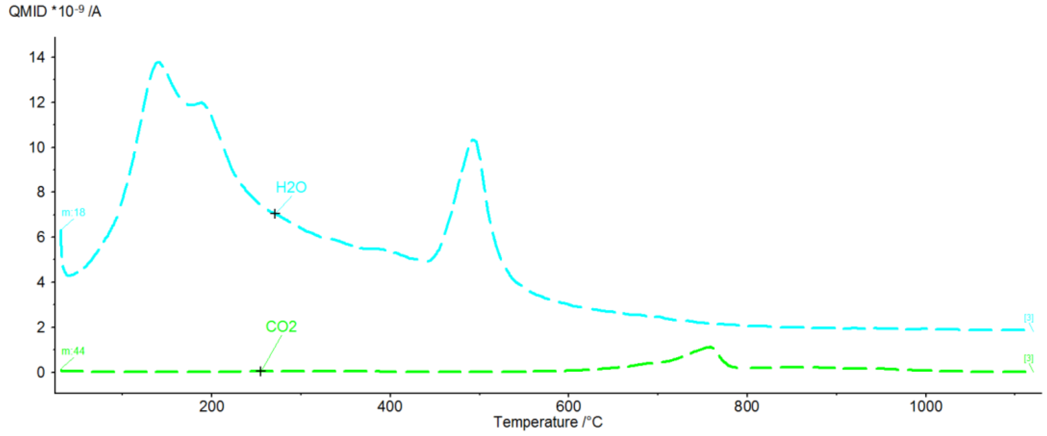

Figure S5. - Sample 5-1. DTA/TG/DTG/EGA thermal curve (the upper panel) and the respective temperature dependent ion current for H_2O and CO_2 release, as indicated (the lower panel).

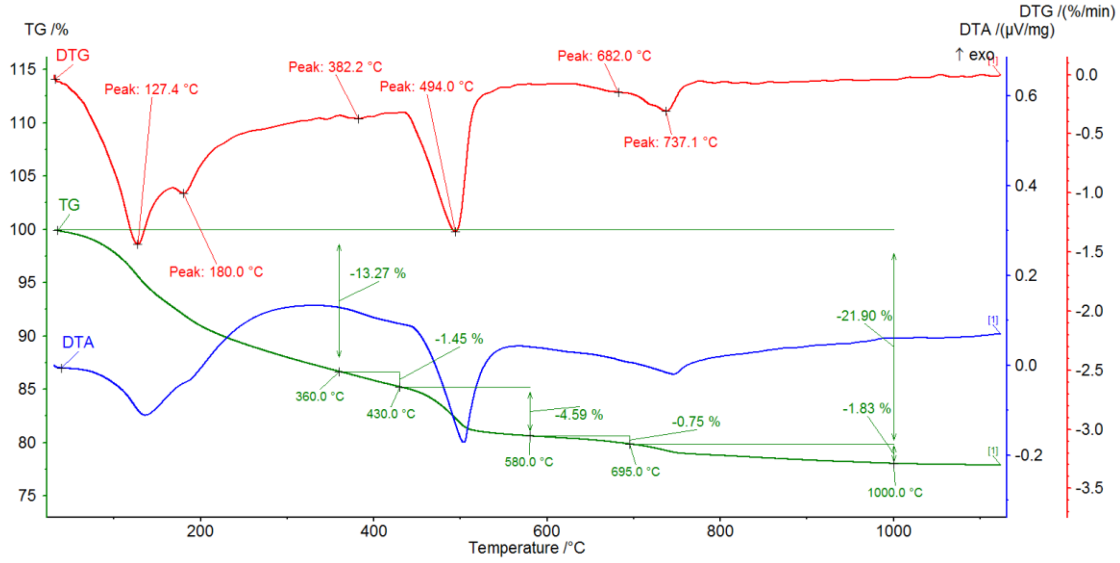

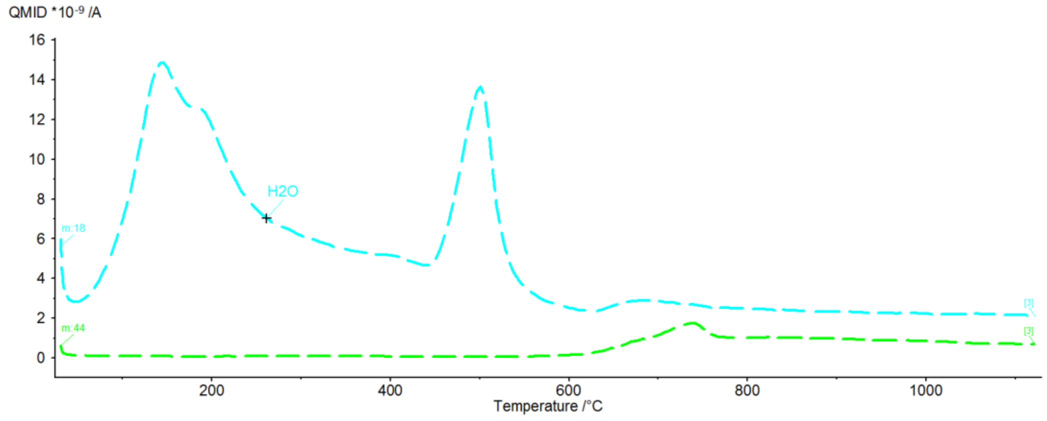

Figure S6. - Sample 6-1. DTA/TG/DTG/EGA thermal curve (the upper panel) and the respective temperature dependent ion current for H_2O and CO_2 release, as indicated (the lower panel).

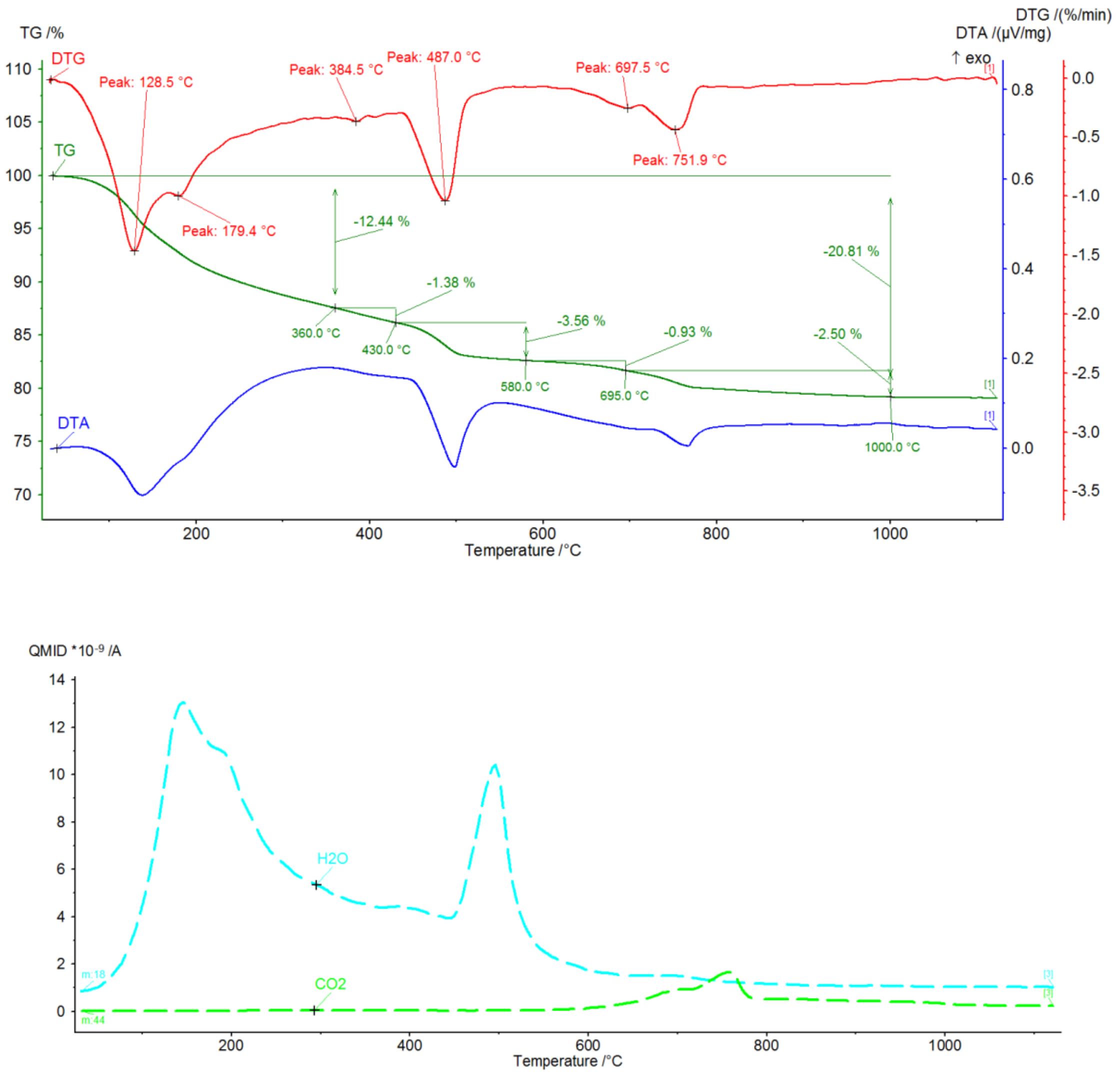

Figure S7. - Sample 7-1. DTA/TG/DTG/EGA thermal curve (the upper panel) and the respective temperature dependent ion current for H_2O and CO_2 release, as indicated (the lower panel).

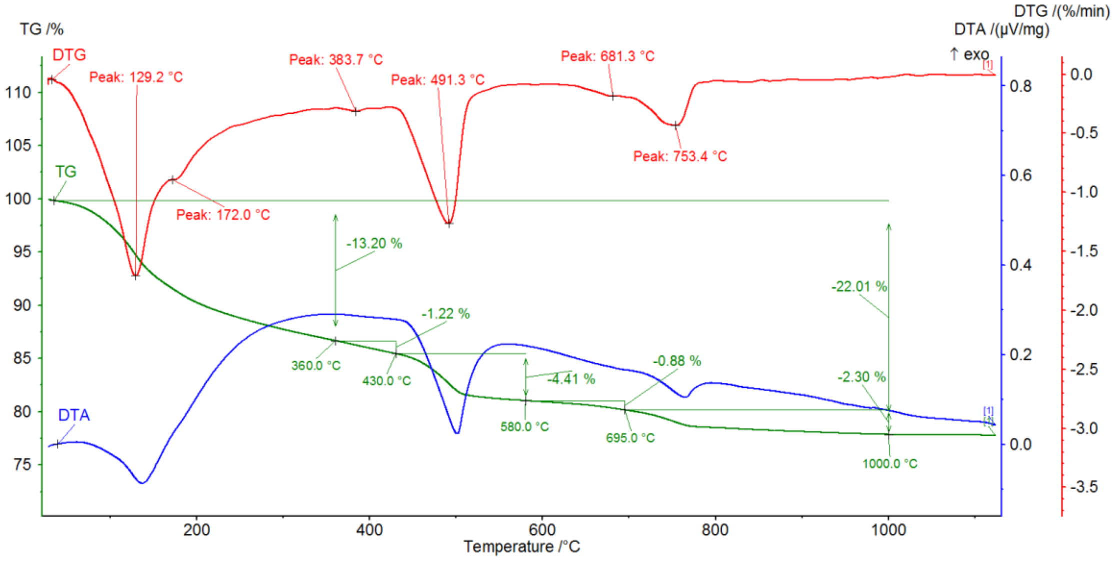

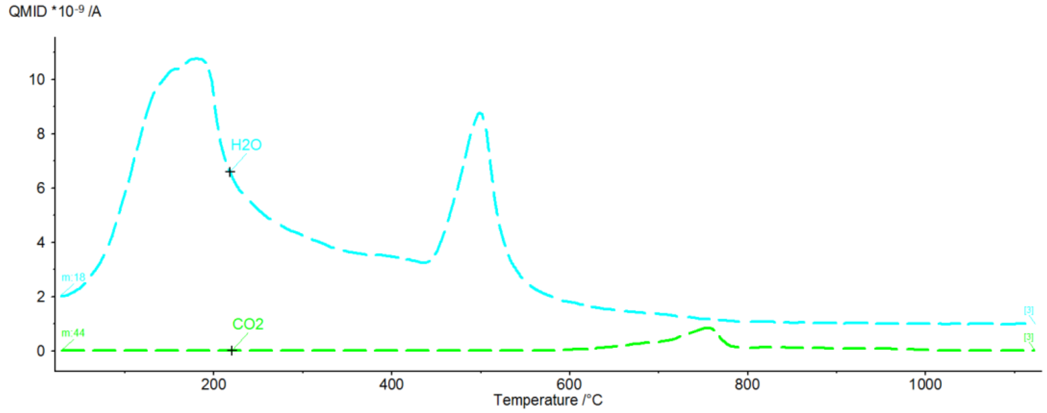

Figure S8. - Sample 1-3. DTA/TG/DTG/EGA thermal curve (the upper panel) and the respective temperature dependent ion current for H_2O and CO_2 release, as indicated (the lower panel).

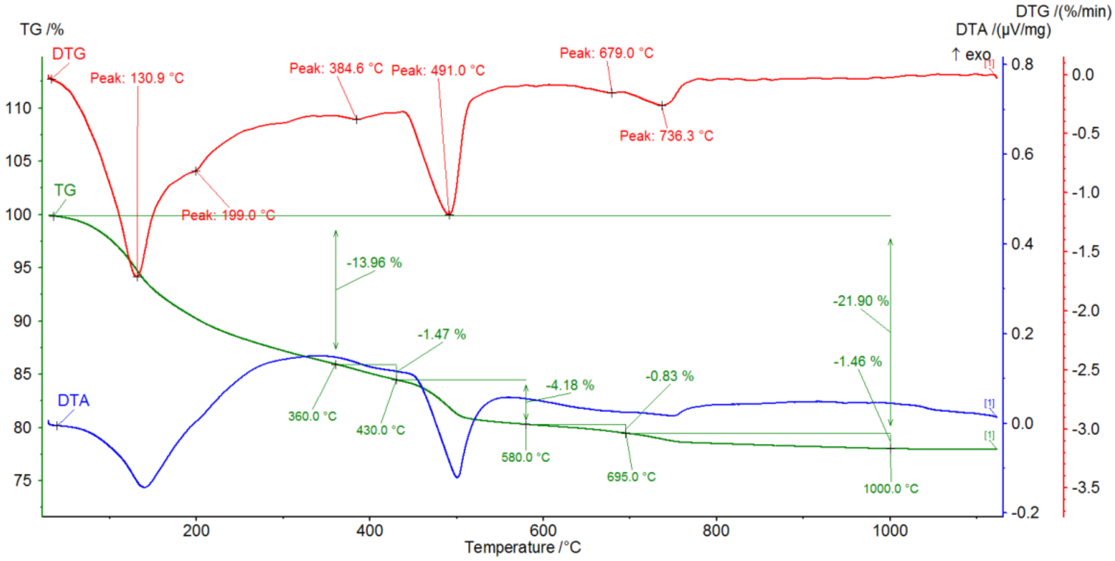

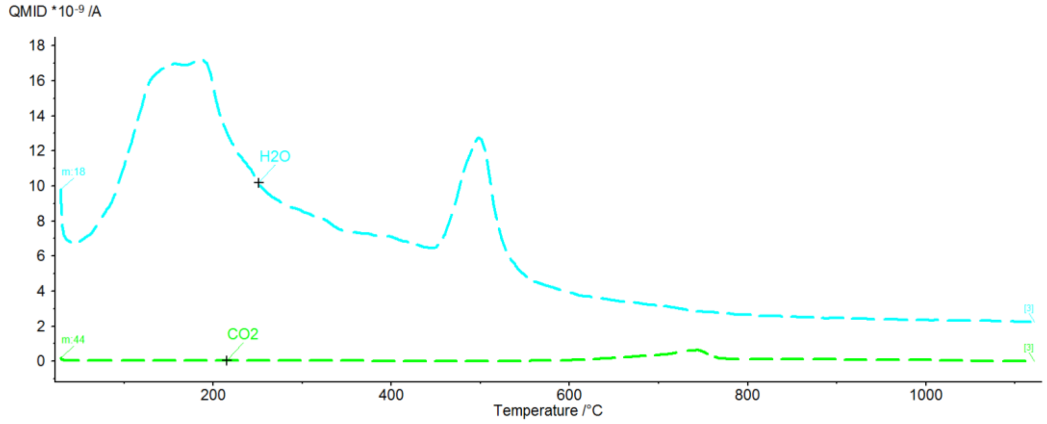

Figure S9. - Sample 2-3. DTA/TG/DTG/EGA thermal curve (the upper panel) and the respective temperature dependent ion current for H_2O and CO_2 release, as indicated (the lower panel).

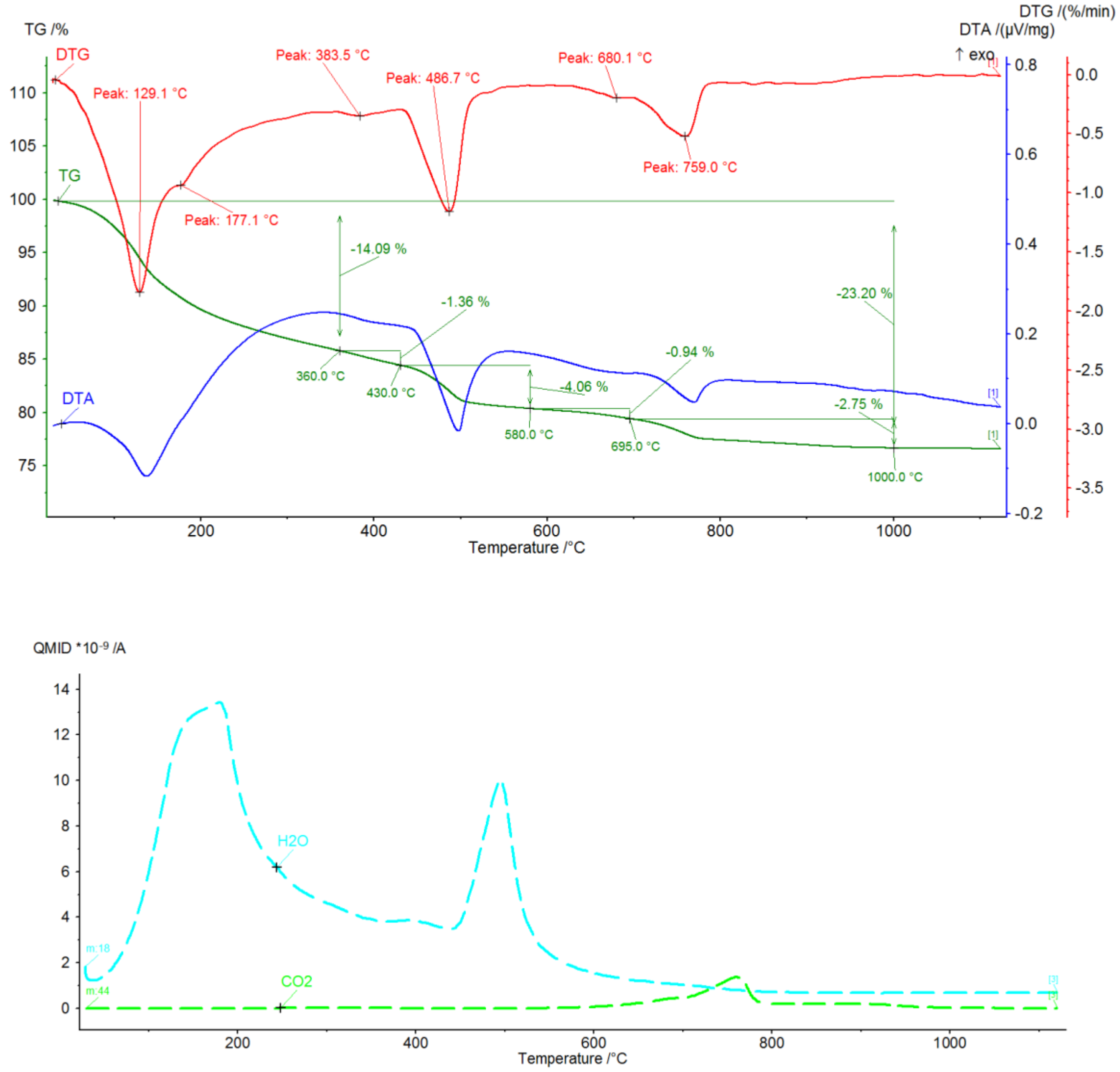

Figure S10. - Sample 3-3. DTA/TG/DTG/EGA thermal curve (the upper panel) and the respective temperature dependent ion current for H_2O and CO_2 release, as indicated (the lower panel).

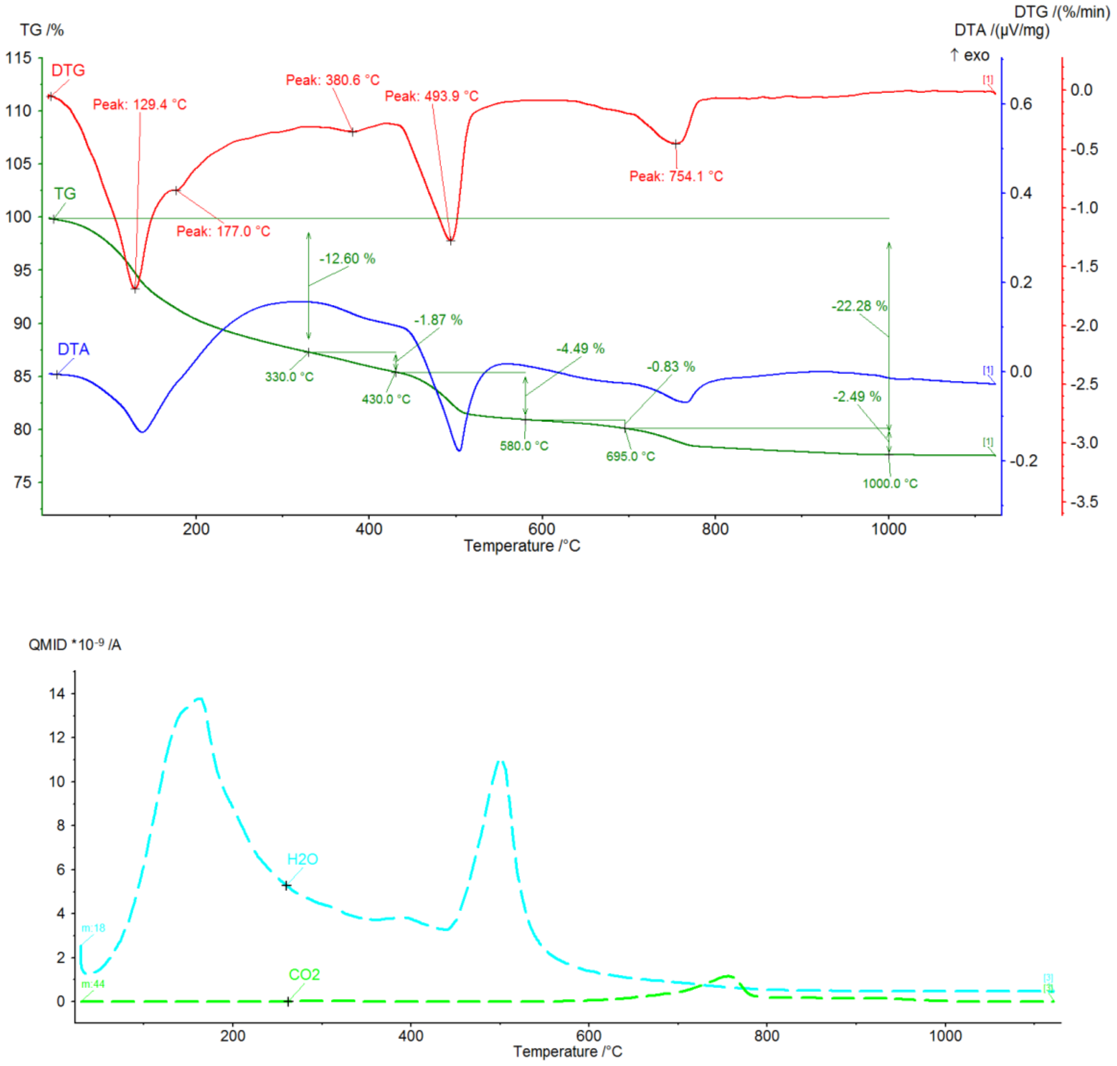

Figure S11. - Sample 4-3. DTA/TG/DTG/EGA thermal curve (the upper panel) and the respective temperature dependent ion current for H_2O and CO_2 release, as indicated (the lower panel).

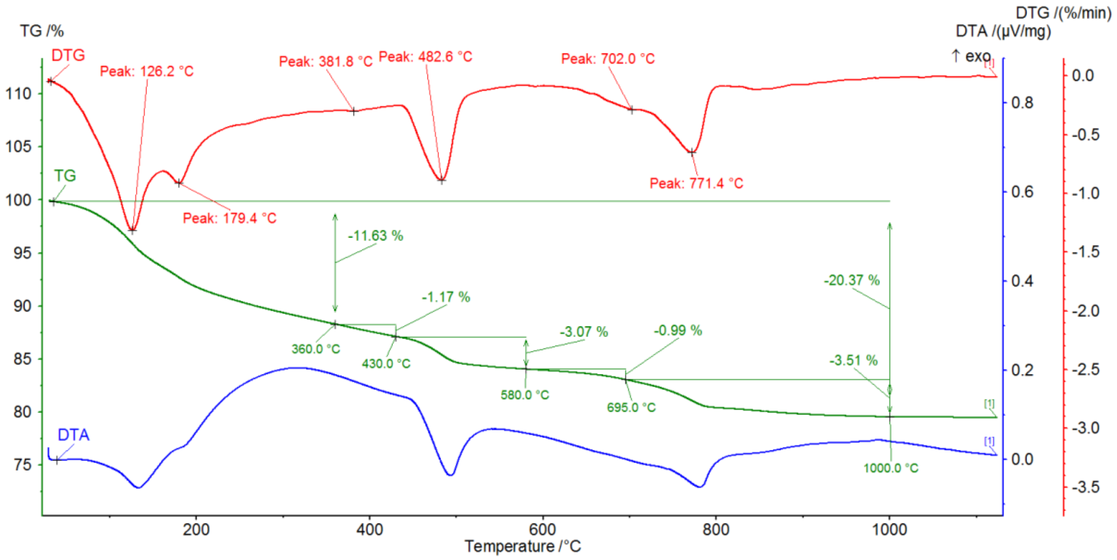

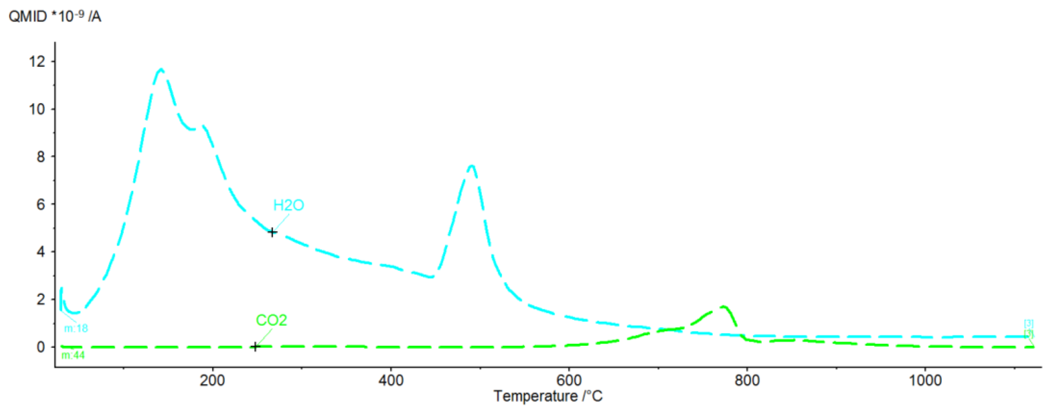

Figure S12. - Sample 5-3. DTA/TG/DTG/EGA thermal curve (the upper panel) and the respective temperature dependent ion current for H_2O and CO_2 release, as indicated (the lower panel).

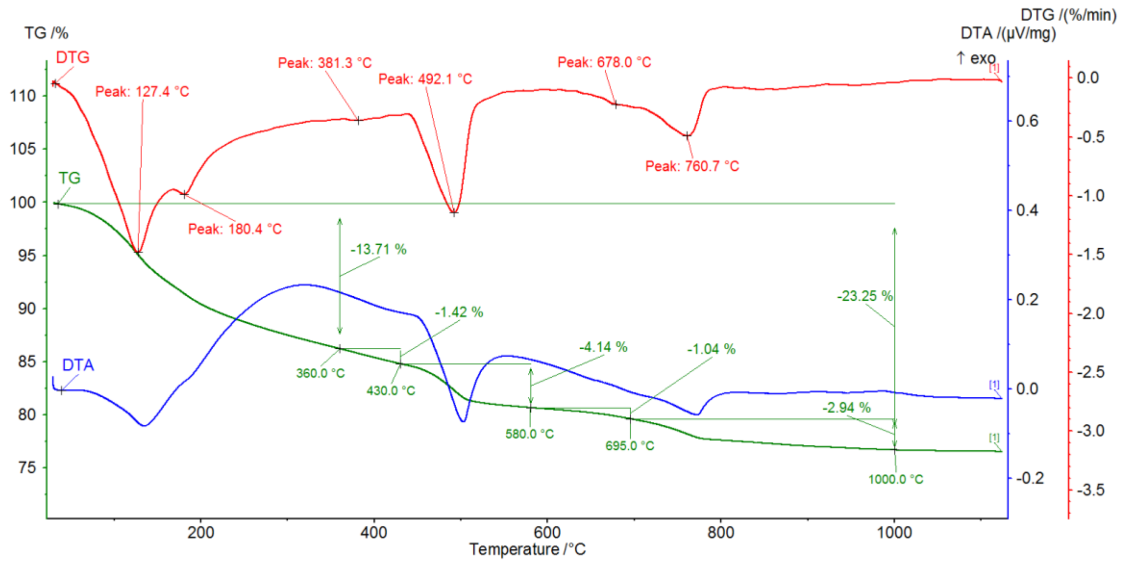

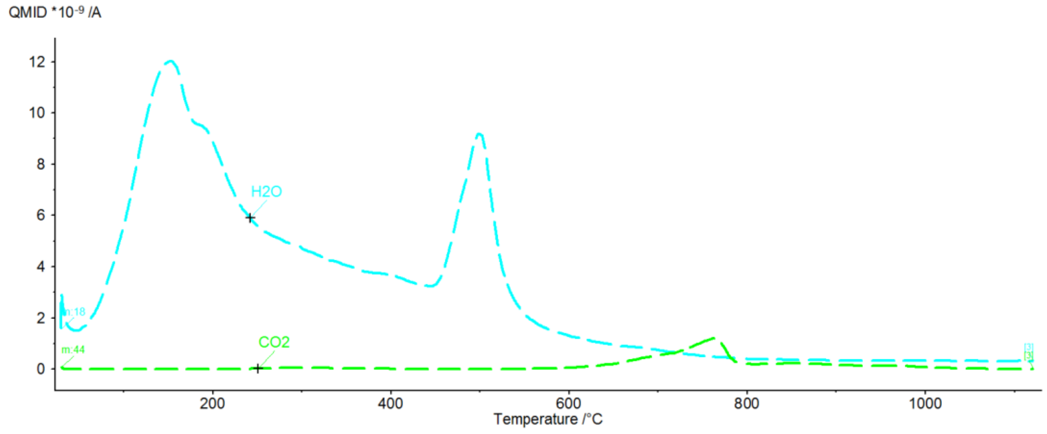

Figure S13. - Sample 6-3. DTA/TG/DTG/EGA thermal curve (the upper panel) and the respective temperature dependent ion current for H_2O and CO_2 release, as indicated (the lower panel).

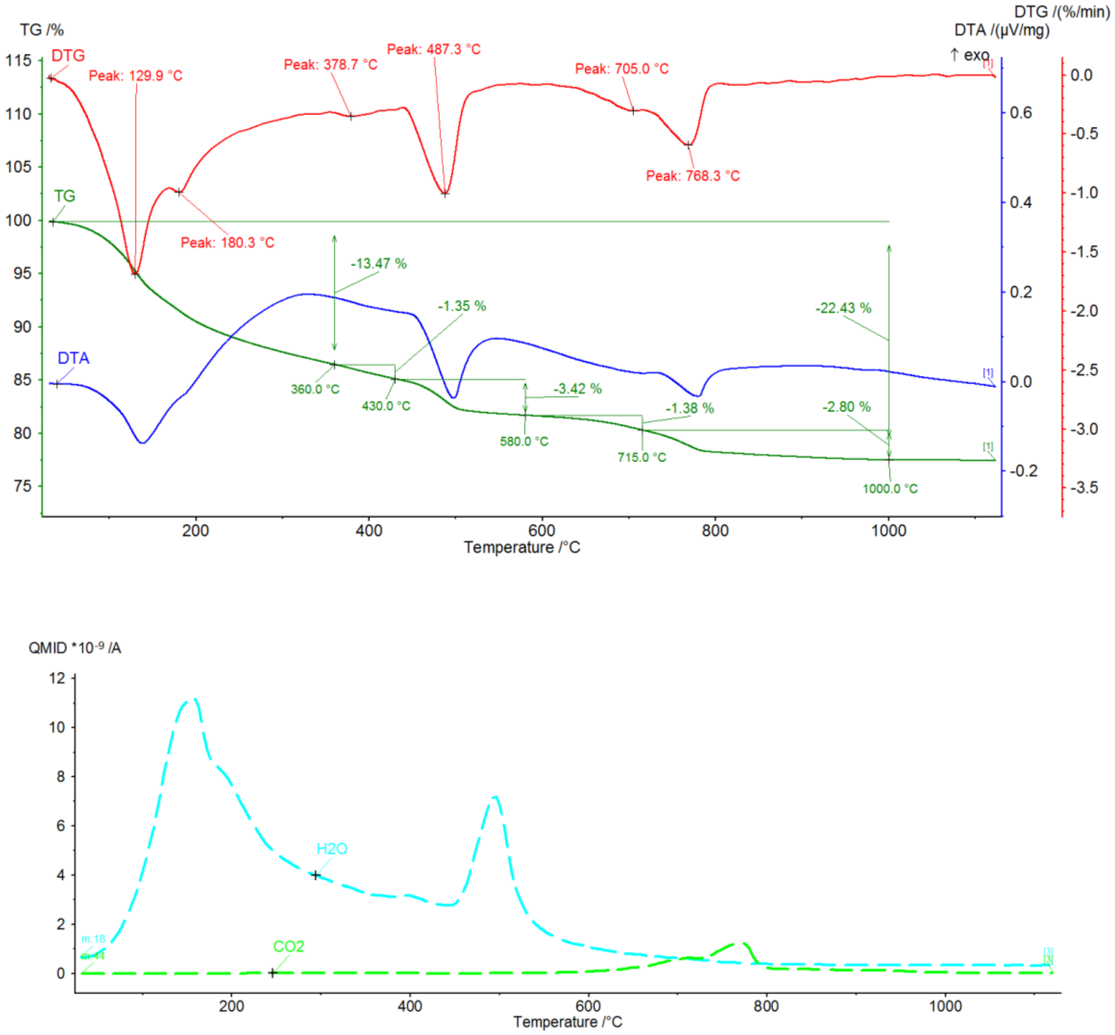

Figure S14. - Sample 7-3. DTA/TG/DTG/EGA thermal curve (the upper panel) and the respective temperature dependent ion current for H_2O and CO_2 release, as indicated (the lower panel).
